# Ischemia-Reperfusion Injury and Ischemic-Type Biliary Lesions following Liver Transplantation

**DOI:** 10.1155/2012/164329

**Published:** 2012-02-29

**Authors:** Raffaele Cursio, Jean Gugenheim

**Affiliations:** ^1^Service de Chirurgie et Transplantation Hépatique, Hôpital l'Archet 2, Centre Hospitalier Universitaire de Nice, Université de Nice Sophia-Antipolis, 151 Route Saint Antoine de Ginestière, B.P. 3079, 06202 Nice Cedex 2, France; ^2^INSERM U526, Equipe 2: Cell Death, Differentiation and Cancer, Centre Méditerranéen de Médecine Moléculaire, Bâtiment Universitaire ARCHIMED, Université de Nice Sophia-Antipolis, 151 Route Saint Antoine de Ginestière, B.P. 2 3194, 06204 Nice Cedex 3, France

## Abstract

Ischemia-reperfusion (I-R) injury after liver transplantation (LT) induces intra- and/or extrahepatic nonanastomotic ischemic-type biliary lesions (ITBLs). Subsequent bile duct stricture is a significant cause of morbidity and even mortality in patients who underwent LT. Although the pathogenesis of ITBLs is multifactorial, there are three main interconnected mechanisms responsible for their formation: cold and warm I-R injury, injury induced by cytotoxic bile salts, and immunological-mediated injury. Cold and warm ischemic insult can induce direct injury to the cholangiocytes and/or damage to the arterioles of the peribiliary vascular plexus, which in turn leads to apoptosis and necrosis of the cholangiocytes. Liver grafts from suboptimal or extended-criteria donors are more susceptible to cold and warm I-R injury and develop more easily ITBLs than normal livers. This paper, focusing on liver I-R injury, reviews the risk factors and mechanisms leading to ITBLs following LT.

## 1. Introduction

After liver transplantation (LT), the incidence of biliary complications, which include a wide spectrum of functional and anatomical abnormalities varies from 10 to 30% [1–3]. These biliary complications lead to an increase of graft dysfunction and patient morbidity and in some cases even to graft loss [4] and retransplantation [5]. They are associated with an increased mortality rate (8 to 15%) [6].

Liver ischemia-reperfusion (I-R) injury during transplantation occurs at different periodes [7]. The first, after liver explantation from the donor and storage on ice at 0° to 4°C, is a variable but generally long period of cold ischemia. The time of vascular anastomosis, when the liver is removed from ice until its implantation in the recipient, represents the second, relatively shorter period of warm I-R injury. In this period of ischemia, the liver warms slowly up to a temperature of 12.5°C during the realization of suprahepatic cava and portal vein anastomoses, and to a temperature of 34°C, once hepatic artery anastomosis is performed [8]. Now the liver is fully revascularized and graft temperature stabilizes. Normothermic reperfusion of the implanted liver with the recipient's blood at 37°C delineates the third period.

Liver ischemia-reperfusion injury following LT causes up to 10% of early transplant failures and can lead to acute and chronic rejection [9]. Moreover, liver I-R injury is associated with intra- and/or extrahepatic nonanastomotic biliary strictures following liver transplantation [4, 10–13].

The ischemic injury itself, a localized process of cellular metabolic disturbances, results from glycogen consumption, lack of oxygen supply and adenosine triphosphate (ATP) depletion [14]. Reperfusion, which consists of initial phase injury (within 2 h after reperfusion) and late phase injury (6–48 hours after reperfusion), aggravates the cellular injuries caused by the ischemic period [9, 15–17].

Although all types of ischemia share common mechanisms cold ischemia of the liver is characterized mainly by injury to sinusoidal lining cells and disruption of the microcirculation, whereas warm ischemia results primarily in Kupffer cell (KC)-derived cytotoxic molecule-mediated hepatocellular injury [17–19].

Liver I-R injury during transplantation involves necessarily the peribiliary plexus resulting in endothelial cell activation, which triggers a cascade of events leading to microvascular thrombosis, microcirculatory disturbances and again ischemia [10, 20]. Stricture formation, biliary apoptosis, necrosis, and cholangitis are the results and may even lead to progressive graft failure. Indeed, it seems that cholangiocytes are more sensible to the ischemic insult than the liver parenchyma [10].

## 2. Anatomy and Blood Supply of the Biliary System

The human biliary system is divided into extrahepatic and intrahepatic bile ducts and is lined by biliary epithelial cells (or cholangiocytes). The classical extrahepatic biliary anatomy consists of a right and left hepatic duct draining the right and left liver lobes, respectively [21–23]. The fusion of the right and left hepatic ducts gives rise to the common hepatic duct (choledochus) [21–23]. The intrahepatic bile ducts are further sub-divided into large and small bile ducts [24–26]. They represent that part of the biliary tree proximal to the confluence of the hepatic ducts [27] extending from the canals of Hering to the large extrahepatic ducts [24–26]. Small ductules that are lined by 4-5 cholangiocytes have a basement membrane, tight junctions between cells, and microvilli projecting into the bile duct lumen [25]. In larger bile ducts cholangiocytes too are progressively larger and more columnar in shape. Ten to twelve cholangiocytes line a larger bile duct [28, 29]. The vascular plexus of the biliary system is composed of branches arising directly from the right and left hepatic arteries (and accessory hepatic arteries when present) and their segmental branches and indirectly from the gastroduodenal artery via the arteries supplying the common bile duct [21–23]. This peribiliary vascular plexus is arranged around the extra- and intrahepatic biliary tree in normal liver [25]. The peribiliary vascular plexus delivers blood to the sinusoids both through lobular branches and through peribiliary branches into the portal vein [25]. In very small portal spaces a small capillary, representing the terminal branches of the hepatic artery, can continue the course of the arteriole and eventually run into the sinusoids [25]. In large portal spaces, the peribiliary vascular plexus is an anastomotic network between short collateral arterioles arising from the same arterial branches. Since the blood flows in the opposite direction to the bile (from the large towards the small vessels), the peribiliary vascular plexus presents a countercurrent stream [25, 30]. The veins draining the surface of the bile ducts follow closely the arterial plexus and drain into the marginal veins. The marginal veins end in subcapsular capillaries related to the hilum of the liver [31].

## 3. Classification of Biliary Complications Following LT

Bile duct strictures following LT have been classified as anastomotic strictures (ASs) and nonanastomotic biliary strictures (NAS) [32]. ASs are isolated strictures at the site of the bile duct anastomosis (choledochocholedochostomy or choledochojejunostomy reconstruction), while NASs concern strictures located in both, the extrahepatic and intrahepatic biliary system of the liver graft [32]. NASs occur after hepatic artery thrombosis (HAT), but also with an open hepatic artery [32]. NASs with an open hepatic artery represent a separate entity and are generally referred to as ischemic-type biliary lesions (ITBLs) [32, 33]. ITBLs were also termed as “ischemic-type biliary complications or ITBC” [3], “ischemic cholangitis” [34] and “ischemic cholangiopathy” [35]. NAS were subclassified according to their etiology: (a) NAS secondary to miaroangiopathic injury (hepatic arterial thrombosis or stenosis), (b) ITBLs secondary to microangiopathic injury (preservation injury, prolonged cold and warm ischemia times, donation after cardiac death, and prolonged use of dopamine in the donor), and (c) ITBLs secondary to immunogenetic injury (ABO incompatibility, rejection, autoimmune hepatic disease, CMV infection, and chemokines polymorphisms) [36].

## 4. Incidence and Risk Factors of ITBLs Following LT

The incidence of ITBLs following LT is 5–15% [33]. This great variability may be partially due to the different definitions used for ITBLs. Although most ITBLs secondary to ischemic lesions occur within 1 year after the transplantation, their prevalence continues to increase with time after liver transplantation [5]. ITBLs appearing more than 1 year after transplantation are mainly related to immunological causes [5].

Risk factors involved in the development of ITBLs are old donor age [36, 37], prolonged cold and warm ischemia times [4, 10–12], non-heart-beating donors (NHBD) [38, 39], graft steatosis [40, 41], some graft perfusion methods [42], high viscosity of cold storage solutions [43, 44], prolonged use of dopamine in the donor [45], and posttransplant liver cytolysis and cholestasis due to I-R injury [46, 47]. In liver transplantation, the increasing gap between the number of patients awaiting an organ and the number of available organs has led to the use of extended-criteria donor (ECD) organs, including organs, which present risk factors mentioned above [48]. As livers from suboptimal donors or ECD are more susceptible to I-R and preservation injury, primary nonfunction (PNF), initial poor graft function (IPGF), delayed graft function (DFG), and also ITBLs are more frequent in these organs [4, 12, 37, 38, 49, 50].

## 5. Pathomechanisms of ITBLs Following LT

ITBLs following liver transplantation result in bile duct destruction and subsequent stricture formation; even a case of sequestration of the bile duct has been described [51]. As shown in Table 1, there are three main interconnected mechanisms causing ITBLs after LT: cold and warm I-R injury [4, 13, 39, 44, 46, 52], injury induced by cytotoxic bile salts [53–57], and immunological-mediated injury [4, 58–76].

## 6. Cold and Warm I-R Injury and ITBLs Following LT (Figures 1 and 2)

Cold ischemic storage of the liver graft and its reperfusion produces injury to the biliary epithelium [2, 13] and is strongly associated with the development of biliary strictures including ITBLs [77].

As Kupffer cells are situated within the lumen of the sinusoid, they are in direct contact with the endothelial surface. From this position when activated by I-R, they release ROS, proinflammatory cytokines, such as tumor necrosis factor alpha (TNF*α*) and oxidants into the circulation as well as directing them to the endothelial layer and the underlying hepatocytes [78]. Although ROSs are essential to cell life at physiological levels, when overproduced they may be responsible of IPGF after LT [79]. Increased production of ROS is associated to reduced basal levels of intracellular glutathione, a principal nonprotein thiol responsible for maintaining intracellular redox status and protecting cells against oxidative injury [80]. Glutathione has an important role in the prevention of cellular ischemia-related oxidative injury during liver preservation by reducing biliary tract cell ROS production [10]. Glutathione present in bile may prevent cholangiocyte injury by counteracting the cytotoxic effects of ROS within the biliary tract [2, 10]. Glutathione is also one of the major determinants of bile acid-independent bile flow [81]. In animals, impaired biliary excretion of glutathione may contribute to the decreased bile flow after cold ischemia [82]. Decreased biliary glutathione excretion is due to impaired transport across the canalicular membrane [82], but also to increased intrabiliary degradation by solubilized *γ*-glutamyltranspeptidase (GGT) [83]. The resulting lower biliary glutathione concentrations diminish the resistence of the cholangiocytes to oxidative stress provoked by I-R [83, 84] and induce cholangiocyte apoptosis [84] through the loss of the antiapoptotic protein B-cell CLL/lymphoma 2 protein (Bcl-2) [85]. Thus, glutathione depletion might explain the intense injury of bile ducts seen in LT [10, 11, 84, 86].

The late or subacute phase of I-R injury is a polymorphonuclear (PMN) leukocyte-dependent process in which the above described ROS generation is associated to cytokine and chemokine expression [78, 87, 88].

The epithelial-lining cells of the biliary system are not only exposed to proinflammatory mediators deriving from intrahepatic sources, but also to those deriving from extrahepatic sources via arterial circulation [89]. These inflammatory mediators promote the invasion of PMNs into the interstitium via the upregulation of adhesion molecules and formation of chemotactic agents [87, 90]. PMNs can penetrate the ductal basal membrane and thus contribute to bile duct injury [91]. Ductal cells are desquamated to the lumen of the bile duct and, together with PMNs, they appear in bile during the first few days after LT [92–94]. There is a clear relationship between postreperfusion hepatic biopsy findings (the degree of PMN infiltration and hepatocellular necrosis of the graft) and biliary complications after liver transplantation, including ITBLs [77].

PMNs and platelets synergistically exacerbate sinusoidal endothelial cell injury by induction of apoptosis during reperfusion. During cell anoxia, cholangiocytes are significantly more resistant to cell death than hepatocytes [10]. This is inverted after reoxygenation of the anoxic cells (which mimics tissue reperfusion), when hepatocytes are more resistant to cell death than cholangiocytes. The rate of ROS formation by cholangiocytes during reoxygenation is greater than in hepatocytes at this moment with concomitant low basal levels of the antioxidant glutathione in cholangiocytes [10]. These findings suggest that bile duct injury after LT is mainly caused during the reperfusion period [10]. Liver reoxygenation upregulates other apoptotic receptor expression than Fas and enhances apoptosis in human biliary epithelial cells [20]. Tumor necrosis factor-related apoptosis-inducing ligand (TRAIL) which binds TRAIL receptor1/death receptor 4 (DR4) and TRAIL receptor2/death receptor 5 (DR5) membrane death receptors can activate apoptosis [95]. Reoxygenation up-regulates DR4 and DR5 expression and enhances TRAIL-mediated apoptosis in human intrahepatic biliary epithelial cells [20].

Human bile epithelial cells [20] do not normally express DR5 [96], but during reoxygenation an even increased DR5 expression of cholangiocytes can be observed [20]. Reoxygenation increases also the activity of caspase-8 and caspase-3 in a TRAIL-dependent manner [20]. Some studies demonstrated an association of a longer warm ischemia time and a marked cholangiocyte apoptosis [20, 97]. Cholangiocyte apoptosis after cold and warm liver I-R is at least partly involved in the pathogenesis of ITBLs after LT [86].

## 7. Intrahepatic Cholestasis and Pathological Effects of Bile Salts Following LT

### 7.1. Intrahepatic Cholestasis

Bile formation requires the coordinated function of hepatocytes and intrahepatic cholangiocytes, which represent 2 to 5% of liver cells [98, 99]. Hepatocytes produce primary or hepatic bile, which percolates through the intrahepatic bile ducts. During this journey, cholangiocytes modify the bile via secretory and absorptive processes that provide additional bile water and alkalinity [100–102]. Cholestasis is an impairment of bile secretion, which results either from a functional defect at the level of hepatocytes (hepatocellular cholestasis) or from an impairment in bile secretion and flow at the level of bile ductules or ducts (ductular/ductal cholestasis) [103]. Intrahepatic cholestasis following liver transplantation is common and generally subclinical [104–107]. However, when severe, cholestasis may be associated with irreversible liver damage, requiring retransplantation [104, 108]. One of the main causes of intrahepatic cholestasis after LT is cold and warm I-R injury [104, 109]. Under normal conditions, bile production requires an active vectorial secretion of biliary constituents from portal blood plasma into bile canaliculi [110]. An intact cytoskeleton is required for bile canalicular contraction, which is based on a pericanalicular web of contractile proteins, actin microfilaments, and cytokeratin intermediate filaments [111] acting as a pump to facilitate bile flow into the intrahepatic canalicular system [112, 113]. The bile canaliculus is one of the liver structures that is early damaged by I-R [105]. This oxidative stress-dependent structural damage contributes to perturbate the bile acid transport during ischemia. The resulting loss of microvilli and the canalicular atony, decrease the bile flow and lead to cholestasis [105–107]. The impairment of bile canaliculi structure following I-R, and postreperfusion biliary complications observed in patients undergoing LT, may be due to an altered reassociation of Ras GTPase-activating-like protein IQGAP1, a regulator molecule of bile canaliculi structure, with the endocytic machinery, particularly with the endocytic multimeric (AP-2) and monomeric (clathrin) adaptors (proteins that mediate the interactions between “address tickets” on cargo proteins and clathrin, as clathrin cannot bind directly to cargo or membranes) [114]. The maintenance of the hepatocyte bile secretion properties would then depend on their ability to rapidly rereform integral adherent junctions and maintain bile canaliculi structure upon reperfusion [114].

Although during 120 min of ischemia or ATP depletion, cell viability and integrity of tight junctions supported by adherent junctions in cholangiocytes were maintained, striking alterations in the secondary structure of their plasma membrane, with decrease of the basolateral interdigitations and apical microvilli have been observed [115]. This reorganization of cholangiocyte membrane domains represents an early event in rat liver ischemia and contributes to impaired vectorial bile duct secretion and postischemic cholestasis [115].

During the ischemic phase failure of the sodium pump or Na(+), K(+)-ATPase [116] leads to intracellular accumulation of Na(+), edema, and swelling of Kupffer cells, sinusoidal endothelial cells, and hepatocytes [117]. Hepatocellular Na(+), K(+)-ATPase is an important driving force for bile secretion and has been localized in the basolateral plasma membrane domain [118, 119]. Bile acid uptake by the hepatocyte is a secondary active transport that is energized by the Na(+) gradient maintained by the Na(+), K(+)-ATPase. Thus, Na(+), K(+)-ATPase appears important in coupling the energy from ATP to transport activity, resulting in so-called bile acid-dependent bile flow [120]. Decreased Na(+), K(+)-ATPase activity following cold and warm I-R results in apoptosis, necrosis, and shedding of biliary tract epithelial cells [121]. Reasons for alterations of Na(+), K(+)-ATPase activity after hypoxia and reoxygenation in the perfused rat liver [122] and after cold and warm I-R in human LT [123], may be direct alteration of the enzyme catalytic subunit and modification of its environment; ROS released from activated Kupffer cells, changes in ATP levels and in membrane lipid fluidity and ionic distribution may also contribute to Na(+), K(+)-ATPase activity disturbances [122]. Moreover, a marked delay of functional recovery in cultured biliary epithelial cells, which was provoked by ATP depletion, induced intrahepatic bile duct injury following I-R [124].

### 7.2. Pathological Effects of Bile Salts

Cholestasis induced by I-R injury is characterized by dilatation of bile canaliculi and loss of microvilli [105] and exposes hepatocytes and cholangiocytes to an elevated concentration of toxic bile acids [125].

Bile formation is an energy consuming process, which is regulated by specific transport proteins situated in the membrane of hepatocytes and cholangiocytes [126]. I-R can induce selective and/or temporary modification of the expression and function of some biliary transporters, leading to abnormal bile composition and to toxic injury to the cholangiocytes [110, 127], as well as to the hepatocytes [98].

The toxic bile composition early after LT, characterized by a low biliary phospholipid/bile salt ratio, is associated with histological signs of injury of the small bile ducts in the liver [51, 54, 56]. The most important apoptotic initiator in cholangiocytes is the Fas receptor/Fas ligand pathway [128]. Human cholangiocytes express Fas receptor [129]. Activated Fas receptor complexes on the plasma membrane cause caspase-8 activation and trigger apoptosis [128]. By liver I-R activated Kupffer cells can potentiate cholestatic injury through the synthesis of the proapoptotic Fas-independent receptor TRAIL [130]. Then, as in a vicious circle, during cholestasis bile acids themselves may initiate or aggravate hepatocellular damage [131]. Toxic hydrophobic bile acids retained in the hepatocytes during cholestasis initiate the generation of ROS metabolites from mitochondria, leading to lipid peroxidation and loss of cell viability [132–134]. The mitochondrial oxidative stress triggers the mitochondrial permeability transition (MPT), resulting in exaggerated mitochondrial cytochrome *c* release and apoptosis [135].

Biliary secretion of HCO_3_(−) prevents the uncontrolled membrane permeation of cytotoxic hydrophobic bile salts by maintaining an alkaline pH near the apical surface of hepatocytes and cholangiocytes [13, 136].

The cholangiocyte “protector” HCO_3_(−) secretion may be disturbed after LT, as I-R results in altered expression of the anion exchanger 2 (AE2) and of the cystic fibrosis transmembrane conductance regulator (CFTR) proteins, which regulate the biliary secretion [137].

Prolonged cold ischemia time during LT is associated to a downregulation of membrane-associated Mucine 1 (Muc1), 3A (Muc3A), and 5B (Muc5B) expression [138, 139]. Mucines are expressed on the apical membrane of the biliary epithelial cells and lubricate and protect these cells from diverse injuries, including injury by cytotoxic bile salts [140]. Decreased expression of Muc1 and Muc3A after LT may favour the development of ITBLs [138].

## 8. Immunologically Mediated ITBLs Following LT

### 8.1. ABO Incompatibility

In the past, liver transplantation across ABO blood group barriers has been discouraged because of multiple complications, particularly acute rejection and biliary complications [58]. However, organ shortage and new developed immunosuppressive agents decreasing humoral rejection have led to an increased use of ABO-incompatible liver for transplantation with acceptable results concerning patient and graft survival rate [65, 141, 142]. Although in children, there is no obvious difference in the outcome of ABO-compatible LT and ABO-incompatible LT, in adults graft survival rate after ABO-incompatible LT is not so satisfactory [65]. Moreover, the incidence of ITBLs after ABO-incompatible LT in adults is much higher than in ABO-compatible LT [60, 65, 67].

ABO blood group antigens are expressed on both, bile duct epithelium and vascular endothelial cells [143, 144]. Donor ABH antigen expression up to 150 days after LT is associated with a high incidence of late, severe biliary strictures (82%), hepatic artery complications (24%), decreased graft survival (44%), and acute cellular rejection [60]. Persisting ABH antigen expression after ABO-incompatible LT is often the consequence of the vascular occlusion. Subsequent ischemic injuries caused by endothelial lesions increase the susceptibility to immunologic injury of biliary cells leading to ITBLs [62, 67, 145]. Preexisting primary sclerosing cholangitis and autoimmune hepatitis are also associated with a higher incidence of ITBLs [75, 76, 146].

### 8.2. Acute and Chronic Rejection

In liver allograft rejection, most tissue damage occurs as a consequence of direct cellular immunologic injury to the bile duct epithelium [147].

Acute cellular rejection, occurring generally within 90 days of LT, concerns 50 to 75% of liver allograft recipients [148]. The targets of activated lymphocytes are donor-derived bile duct epithelial cells and vascular endothelium [147]. Acute rejection is associated with lymphocytic cholangitis, a cytotoxic T-cell-mediated nonsuppurative destructive cholangitis of the small intrahepatic bile ducts that can induce cholestasis [147, 149]. Activated Kuppfer cells migrate into the rejecting liver and release cytokines, resulting in the loss of the Na(+), K(+)-ATPase activity, which plays an important role in bile secretion [119]. Tauroursodeoxycholic acid (TUDCA), a hydrophilic bile acid, can protect from cholestatic and hepatocellular injury by enhancing the secretory capacity of the cholestatic liver cells and by its cytoprotective action against hydrophobic bile salts [150–152]. During allograft rejection, the loss of Na(+), K(+)-ATPase activity, a cotransporter for hepatocyte taurocholate uptake, leads to impaired secretion of TUDCA and results in subsequent cholestatic injury [119]. In patients presenting ITBLs following LT, the response of lymphocyte T helper 1 (Th1) was decreased, while the response of Th2 was increased [153]. Whether these immunological changes were induced by the damage of the bile ducts or occur as an additional damaging factor or are found as an epiphenomen in patients with liver transplant dysfunction remains unclear.

Although some studies did not show that chronic graft rejection was a risk factor for development of ITBLs following LT [154, 155], others demonstrated the association of chronic allograft rejection and development of ITBLs [33, 46, 60, 66–68]. Chronic allograft rejection after LT also termed “ductopenic rejection”, is characterized by ischemic injury and paucity of bile ducts [148]. It occurs within the first year after LT with an incidence rate of 2 to 5% [156]. The zone of the central venous drainage or zone 3 of liver parenchyma is poorest in oxygen concentration and more sensitive to ischemia. During cellular chronic rejection, progressive intimal and subintimal infiltration of second- and third-order branches of the hepatic artery with foam macrophages accompanied by foam cells or obliterative arteritis can result in arterial stenoses and ultimately in ischemic injury to interlobular bile ducts and hepatocytes of zone 3 [104, 157]. However, in the early stages of chronic rejection, the necroinflammatory lesions of interlobular bile ducts and hepatocytes, frequently associated with typical portal inflammatory infiltrates of acute cellular rejection, seem more likely to be the results of direct immune-mediated mechanisms [158].

### 8.3. Gender

Gender-mismatched liver transplant recipients had a higher likelihood of graft failure when compared with gender-matched liver transplant recipients [74]. In male recipients receiving female donor organs there is an increased risk of graft failure compared with a female recipient receiving a liver from a male donor [74]. Moreover a female to male donor/recipient match is associated with late occurrence of ITBLs [76].

### 8.4. Cytomegalovirus (CMV) Infection

The overall rate of CMV infection in liver transplant recipients varies from 30 to 50% [159, 160]. In CMV-infected rat liver allografts undergoing acute rejection there was a significant increase in portal inflammation and more severe bile duct injury compared with CMV-negative liver allografts [161]. In transplanted patients developping ITBLs during CMV infection, histological examination of specimens from bile duct strictures showed CMV inclusions [71]. CMV infection induces injury of endothelial cells of the peribiliary capillary plexus, with subsequent microthrombi formation and insufficient oxygenation of the biliary epithelium, ultimately leading to ischemic injury of bile duct cells and development of ITBLs [13, 72]. CMV infection can damage the bile duct cells in a direct manner by infecting directly biliary epithelial cells and in an indirect manner by immune attack evoked against infected biliary epithelial cells [13].

### 8.5. Chemokine Polymorphism CCR5 Delta 32

Loss-of-function mutation in the CC chemokine receptor 5 (CCR5delta32) has been associated with development of ITBLs following LT [61]. Functional changes in the immune system resulting from CCR-5 delta 32 mutation, which include impaired chemotaxis of regulatory T cells to the site of injury may be responsible [162]. Indeed, a greater risk of developing ITBLs after LT was observed in liver transplant recipients carrying CCR5-delta 32 polymorphism compared with CCR5 wild-type transplant recipients [13, 61, 73].

### 8.6. Metalloproteinase-2 (MMP-2) Polymorphism in Liver Graft Donor and Recipient

A large family of proteolytic enzymes involved in the degradation of extracellular matrix called matrix metalloproteinases (MMPs), secreted by Kupffer and stellate cells [163], are involved in the mechanisms of neutrophil infiltration and in the alteration of liver microcirculation due to the loss of the normal sinusoidal extracellular matrix following cold [164] and warm [165, 166] I-R injury. The activation of one of these MMPs, the metalloproteinase-2 (MMP-2), also called Gelatinase-2, takes place at the cell surface, which confers to this unique MMP a pivotal role in cellular migration during processes requiring the remodelling of basement membranes, the thin extracellular matrices underlying endothelial and epithelial cells [167, 168].

MMPs are subject to complex regulation at multiple levels: gene transcription, proenzyme activation, and inhibition of activity by tissue inhibitors of matrix metalloproteinases (TIMPs) [167, 168]. At gene level, it has been demonstrated that several single nucleotide polymorphisms (SP) in the gene promoter regions of MMPs have an impact on the transcription rate into the cells [169, 170]. The SP C/T transition at position—1306 in the promoter of MMP-2, abolishes the single polymorphism 1 binding site and leads to decreased mRNA transcription and protein expression of MMP-2 [169, 170]. After LT, in association with the–1306 CT genotype of donor and recipient, the serum levels of MMP-2 were decreased in patients that developed ITBLs [75].

MMP-2 CT genotype in both, donor and recipient is strongly and independently related to the development of ITBLs within 4 years after LT [75]. The presence of the MMP-2 CT genotype in donor and/or recipient was found to increase the incidence of ITBLs incidence stepwise from 9% when absent, increasing to 16% when present in either donor or recipient, further increasing to 29% when present in both donor and recipient [75]. These findings indicate that a genetically determined reduced MMP-2 tissue remodelling contributes to the development of ITBLs after LT.

## 9. Extended Donor Criteria and ITBLs

### 9.1. Donor Age and ITBLs

As old livers are more susceptible to warm and cold I-R injury than young livers [117–172], donor age seems to be a relevant risk factor in the development of ITBLs after LT [4, 12, 36, 37, 76, 173].

The activation of peroxisome proliferators-activated receptor gamma (PPAR*γ*), which belongs to the hormone nuclear receptor superfamily [174], is significantly reduced in old mice compared to young mice [175]. During liver ischemia, its activation is suppressed [176]. In old mice, PPAR*γ* activation significantly improves liver I-R injury [176] by modulating inflammatory response and apoptosis [177].

Different from young livers, the initiation of apoptosis by nonparenchymal cells in older livers is increased and is driven by the enhanced release of TNF*α* [171]. TNF*α* is a cytokine mainly released by activated Kupffer cells following liver I-R [19, 178, 179]. Aging may directly affect Kupffer cells resulting in TNF*α* release [180, 181] and apoptosis [171]. Apoptosis is a highly regulated ATP-requiring form of cell death [182]. Despite decreased ATP levels and reduced hepatic mitochondrial function in older livers [117], apoptosis seems to be a predominant feature of liver cell death following ischemic injury in these old livers [171]. Lower ATP content in older liver does not directly affect the apoptotic cascade but facilitates the activation of apoptotic mediators and inhibits survival mechanisms [171].

### 9.2. Cold Ischemia Time and ITBLs

Prolonged cold ischemia time is an independent risk factor for liver preservation injury, even more so than donor age [183]. Cold graft preservation for more than 14 h has been associated with a two-fold increase in preservation injury, resulting in biliary stricures and decreased graft survival [183–185]. Accordingly, the risk of graft loss increases by 1% for each additional hour of cold ischemia [186]. Although several studies failed to show a correlation between the incidence of ITBLs and cold ischemia time [44, 68, 155, 173, 187, 188], in other studies this incidence was increased after a prolonged cold ischemia time [2, 12]. After a cold ischemia time less than 13 hours the percentage of ITBLs was 7%, whereas the percentage increased to 52% when the cold ischemia time was longer than 13 hours, and to 69% if it was longer than 15 hours [2, 12]. In a recent large retrospective study with an overall incidence of post liver transplantation ITBLs of 3.9% [12], 10 hours of cold ischemia time turned out to be the threshold that should not be excessed in order to avoid ITBLs, [12].

### 9.3. NHBDs and ITBLs

In liver transplantation, the use of NHBDs has been introduced in order to expand the organ donor pool [189]. However, the addition of donor warm ischemia time to the subsequent cold preservation time and warm reperfusion injury negatively impacts graft function following LT [190].

Compared with donation after brain death (DBD), livers from NHBD inevitably sustain a period of warm ischemia from circulatory arrest until start of preservation, resulting in ischemic injury with higher risk of biliary complications including ITBLs [39]. Also the incidence of IPGF, PNF, acute and chronic rejection, and retransplantation is higher with NHBD [190–194].

In liver transplantation, the overall rate of biliary complications is 29% (range: 11%–53%) for NHBD and 17% (9%–22%) for DBD recipients [190]. The ITBL rate is 16% (8%–38%) for NHBD recipients and 3% (0%–8%) for DBD recipients [39, 52, 190, 192–195]. ITBLs occur within 30 days in NHBD and about 3 months after transplantation in DBD grafts [4].

In NHBD, low blood flow during the period of hypotension after tracheal extubation and no blood flow during the period between cardiac arrest and organ recovery result in formation of microthrombi that obstruct the capillaries and limit liver perfusion [196]. Inadequate flush of these capillaries leads to suboptimal cold preservation and subsequently to exacerbated ischemic injury [196]. In a pig NHBD model of liver transplantation, prolonged warm ischemia time resulted in a high biliary salt-to-phospholipid ratio, which contributes to the development of ITBLs [51].

### 9.4. Graft Steatosis and ITBLs

Steatosis of the liver is considered pathologic when the hepatic fat content, consisting mainly of triglycerides, exceeds 5% of the actual wet weight of the liver [197]. Hepatic steatosis is present in approximately 20% of liver donors, and 5-6% of cadaveric livers are discarded due to steatosis [198]. Liver steatosis is histologically classed as “macrovesicular” when the hepatocytes are distended by a single large fat vacuole that displaces the nucleus to one side of the cell and as “microvesicular” when multiple small droplets finely are dispersed in the cytoplasm without nuclear displacement [197]. More than 30% of macrovesicular steatosis on donor liver biopsy is an independent risk factor for allograft loss at one year along with other elements of the donor risk index [199]. Early biliary complications seems to be associated with moderate macrovesicular steatosis [200, 201]. In a recent study, the time interval between portal and arterial reperfusion and macrovesicular steatosis of the graft of more than 25% revealed to be significant predictors of biliary complications [40, 41]. At Univariate analysis macrosteatosis of more of 25% of the graft is the only independent risk factor predicting biliary complications after liver transplantation [40, 41]. The increased susceptibility of the steatotic liver to I-R injury is due to the perturbation of both, blood flow microcirculation and changes in the cells [202]. Brain death of the liver donor may amplify the adverse effects of preexisting steatosis by inducing hypotension, and reducing portal venous and hepatic microcirculation [203, 204].

## 10. Storage Solutions and Perfusion Methods of the Liver Graft and ITBLs

### 10.1. Graft Perfusion and ITBLs

Although approximately 75% of the total liver blood flow is provided by the portal vein, the hepatic artery supplies approximately 50% of the oxygen consumed by the liver in physiologic conditions [205]. There are two main methods for revascularization of the liver graft: sequential and simultaneous revascularization [42]. In the first method, sequential revascularization, the graft is first reperfused via either the portal vein or the hepatic artery (anterograde reperfusion), or via the inferior vena cava (IVC) (retrograde reperfusion) with subsequent reconstruction of the remaining vessels. In the second method, simultaneous revascularization, the graft is reperfused simultaneously via the portal vein and the hepatic artery. The sequence of graft reperfusion may be relevant for the development of ITBLs, particularly in grafts from ECD [42]. Liver transplantation standard technique involves initial blood perfusion by the portal vein to shorten the anhepatic period and graft rewarming *in situ*. In this period, the graft is exclusively perfused through the portal vein for at least 10 min until the realization of the hepatic arterial anastomosis [40]. The delay of rearterialization in sequential revascularization is associated with more pronounced microvascular disturbances and subsequent graft dysfunction [206]. Indeed rearterialization of the graft during liver transplantation causes an increased volumetric blood flow within the sinusoids called “reactive hyperemia” [207]. A long interval between portal and arterial reperfusion of the liver, in case of sequential revascularization, is associated to a higher incidence of biliary complications following DBD LT [40]. Simultaneous revascularization elicits a remarkable improvement in oxygen tension and maintenance of tissue ATP, compared to sequential revascularization [208]. The disadvantage of simultaneous revascularization is the prolongation of warm ischemia time and the anhepatic phase, which can be detrimental to postoperative graft function and survival [184, 209].

Whether simultaneous revascularization is better than sequential revascularization remains unclear [42, 207, 210, 211].

In some retrospective studies, the incidence of ITBLs in patients who underwent simultaneous revascularization of the graft [45, 212] was lower compared to patients who had sequential revascularization [42, 211]. Particularly in a recent study, simultaneous revascularization resulted in a minor incidence of ITBls compared to sequential revascularization (none versus 26%, resp.) [213], suggesting that simultanous revascularization may be more suitable to protect the integrity of the intrahepatic biliary tree [213].

Retrograde perfusion of the liver graft via the vena cava, followed by anterograde sequential reperfusion of the portal vein and the hepatic artery, decreases liver I-R injury and IPGF [210]. However, on the biliary epithelium or other cells of the biliary tract retrograde reperfusion has detrimental effects with an increased risk of ITBLs [210]. Improvement in flushing the microscopic biliary vasculature and possibly preventing microvascular thrombosis in the biliary tree may be obtained by adding the high-pressure aortal perfusion technique to the main graft perfusion methods, [66] and additional arterial back-table pressure perfusion [36]. These graft perfusion methods seem to reduce the rate of ITBLs following LT [36, 66].

### 10.2. ITBLs: Importance of Portal Venous Blood Flow

The blood supply to the biliary tree is almost solely arterial, with no significant contribution from the portal vein in physiological conditions [31, 214, 215]. However, some support the hypothesis that the peribiliary vascular plexus is not only sustained by blood from the hepatic artery as traditionally reported, but also by blood from the portal vein [216]. The hepatic artery is in essence an end artery for the donor biliary tree, as collaterals from the lower extrahepatic biliary tree are interrupted in the process of liver procurement and transplantation. In case of hepatic artery thrombosis, new collateral vessels can form and limit additional biliary stricture formation [155]. As ITBLs occur in the absence of hepatic artery thrombosis, it has been suggested that the portal venous blood flow has an important impact on the pathogenesis of ITBLs after liver transplantation [216]. In a recent study, patients with partial portal vein thrombosis and intact hepatic arterial blood supply developed ITBLs in the hepatic segments affected by portal vein thrombosis [216]. In many cases of hepatic artery thrombosis it seems that the portal perfusion maintains hepatocytes [216]. Thus, the contribution of the portal blood flow to the biliary microcirculation is not negligible and a compromised portal venous blood supply can predispose to the development of ITBLs [216].

### 10.3. Static Cold Storage Solutions of the Graft and ITBLs

Liver preservation techniques do influence the graft quality [9]. Static and dynamic preservation are the two current methods of liver preservation in LT [217]. Static preservation means simple cold storage while dynamic preservation comprises hypothermic machine perfusion, normothermic machine perfusion, and oxygen persufflation [217]. Until today, only static cold storage preservation is clinically approved for liver transplantation in humans [217].

Cold preservation injuries to the biliary tract of the donor liver were decreased by efficient flushing of the biliary tract in animals [218, 219] and in humans [220, 221]. In a recent study, an effective biliary flush reduced the effects of bile salt toxicity to the epithelium, reduced cell edema, prevented cell acidification, and provided adequate ATP precursor substances, resulting in reduction of biliary cold preservation injuries [222]. Generally, static cold storage UW solution is used for organ preservation [223], however the histidine-tryptophan-ketoglutarate (HTK) static preservation solution has started to compete with UW [224] and is now mainly used in deceased donor liver transplantation (DDLT) in Europe and North America and in living-related liver transplantation (LRLT) in Japan and Hongkong [43, 221, 225–233]. UW cold storage solution has more hepatocytoprotective effects than HTK cold storage solution [234–236], but its viscous nature may hinder an efficient flushing of the small bile duct capillaries, so residual bile can crystallize and obstruct capillary ducts, thus aggravating the cold ischemic insult to the epithelial biliary cells [237, 238]. HTK cold storage solution has the same viscosity as water and its average velocity is three times greater than UW solution under the same perfusion pressure [221]. The time of liver cooling with HTK cold storage solution is shorter and improves the perfusion of the biliary vascular plexus resulting in reduced biliary tract preservation injury [36, 220, 221, 239, 240]. Also the lack of macroaggregate formation of adenosine crystals and the absence of plastic byproducts in HTK solution, responsible for occlusion of small capillaries, which exacerbates small bile duct ischemia following reperfusion, contribute to the beneficial effects of HTK cold storage solution [237, 238]. HTK cold storage solution may be used particularly in livers with existing I-R injury, with high risk of I-R injury, or with biliary injury such as ECD organs [221, 223, 241]. A combined use of both cold storage solutions, HTK with its low viscosity and UW with its hepatocytoprotective effects, may have additional benefits for the biliary system [219, 242]. Thrombolytic agents as urokinase [243], which may help flushing the microscopic biliary vasculature, were employed to prevent microvascular thrombosis in the biliary tree [244].

As there are no standardized guidelines regarding the methods of liver graft perfusion in terms of solution type, amount of solution, route of perfusion, perfusion pressure, and the time of perfusion, adequately powered randomized clinical trials with long follow-up periods are needed to evaluate the long-term impact on warm and cold I-R injury and induction of ITBLs after LT.

## 11. Conclusion

The main pathomechanisms leading to ITBLs following LT are cold and warm I-R injury, stagnation of cytotoxic bile salts and changes in bile composition, and immunological mechanisms. These mechanisms are mutually connected, one inducing or reinforcing the other, that it may be difficult sometimes to settle the “culprit”. Besides, knowledge of these mechanisms remains superficial and in the beginnings. Naturally, that goes too for the possibilities of ITBL prevention and treatment. Until sustained progressions are not made in the field of ITBL research, the only way to keep the incidence of ITBLs after LT as low as possible is to reduce as much as possible their risk factors.

## Figures and Tables

**Figure 1:**
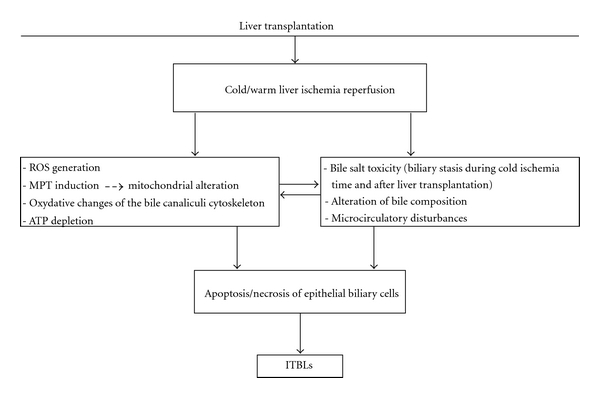
Cold and warm liver I-R leading to ITBLs following LT. ROS: reactive oxygen species; MPT: mitochondrial permeability transition; ATP: adenosine triphosphate.

**Figure 2:**
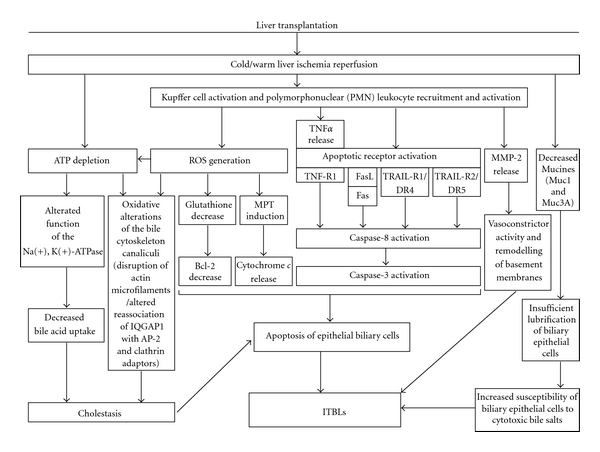
Role of Kupffer cells and PMNs in cold and warm ischemia leading to the development of ITBLs following LT. The epithelial lining of the biliary tree is exposed not only to proinflammatory mediators derived from extrahepatic sources, via arterial circulation, but also to proinflammatory mediators derived from intrahepatic sources, such as inflammatory cells or Kupffer cells. These inflammatory mediators promote the invasion of PMNs into the interstitium. PMNs then penetrate the ductal basal membrane and contribute to bile duct injury. Thus the main event injury seems to be activation of Kupffer cells and recruitment and activation of PMNs leading to apoptosis of epithelial biliary cells. PMN: polymorphonuclear neutrophils; ROS: reactive oxygen species; ATP: adenosine triphosphate; Na(+), K(+)-ATPase: sodium pump; TNF*α*: tumor necrosis factor alpha; TNF-R1: tumor necrosis factor receptor 1; MPT: mitochondrial permeability transition; Bcl-2: B-cell CLL/lymphoma 2 protein; IQGAP1: regulator molecule of bile canaliculi structure; AP-2: endocytic multimeric adaptor; Clathrin: endocytic monomeric adaptors; FasL: Fas ligand; TRAIL-R1/DR4: tumor necrosis factor-related apoptosis-inducing ligand (TRAIL) which binds TRAIL-receptor1/death receptor 4 (DR4); TRIAL-R2/DR5: tumor necrosis factor-related apoptosis-inducing ligand (TRAIL) which binds TRAIL-receptor2/death receptor 5 (DR5); Muc1: mucine 1; Muc3A: mucine 3A; MMP-2: metalloproteinase-2.

**Table 1:** Pathomechanisms leading to ITBLs after liver transplantation.

Type of injury	
Ischemia reperfusion related	

(i) Warm ischemia in the donor	
(ii) Prolonged cold ischemia time	
(iii) Reperfusion injury	
(iv) High viscosity of cold preservation solutions	
(v) Warm ischemia during graft implantation	
(vi) Microcirculatory disturbances in the peribiliary capillary plexus	

Bile salts related	

(i) Cytoprotective hydrophilic bile salts (decreased after liver transplantation)	
(ii) Cytotoxic hydrophobic bile salts (accumulated after liver transplantation)	
Insufficient flush out of bile from the bile ducts during liver transplantation	
High biliary bile salt/phospholipid ratio after liver transplantation	
Impaired vectorial bile duct secretion with intracellular accumulation of bile salts in cholangiocytes	
Impaired biliary secretion of the protecting cholangiocyte factor HCO3(−)	

Immune mediated	

(i) ABO-incompatible liver transplantation	
(ii) Acute rejection	
(iii) Chronic rejection	
(iv) Gender (female liver transplanted in male recipient)	
(v) Cytomegalovirus (CMV) infection in the graft	
(vi) Chemokine polymorphism in graft recipients (CC receptor 5 delta 32)	
(vii) Preexisting autoimmune disease of the graft	
Primary sclerosing cholangitis	
Autoimmune hepatitis	
(viii) Metalloproteinase (MMP) polymorphism in donor and recipient graft	
MM P-2 genotype polymorphism	

## References

[1] Lewis WD, Jenkins RL (1994). Biliary strictures after liver transplantation. *Surgical Clinics of North America*.

[2] Sanchez-Urdazpal L, Gores GJ, Ward EM (1992). Ischemic-type biliary complications after orthotopic liver transplantation. *Hepatology*.

[3] Sanchez-Urdazpal L, Gores GJ, Ward EM (1993). Diagnostic features and clinical outcome of ischemic-type biliary complications after liver transplantation. *Hepatology*.

[4] Guichelaar MMJ, Benson JT, Malinchoc M, Krom RAF, Wiesner RH, Charlton MR (2003). Risk factors for and clinical course of non-anastomotic biliary strictures after liver transplantation. *American Journal of Transplantation*.

[5] Verdonk RC, Buis CI, van der Jagt EJ (2007). Nonanastomotic biliary strictures after liver transplantation, part 2: management, outcome, and risk factors for disease progression. *Liver Transplantation*.

[6] Wojcicki M, Milkiewicz P, Silva M (2008). Biliary tract complications after liver transplantation: a review. *Digestive Surgery*.

[7] Cursio R (2010). Caspase inhibition in liver transplantation: from basic research to clinical studies. *HPB*.

[8] Villa R, Fondevila C, Erill I (2006). Real-time direct measurement of human liver allograft temperature from recovery to transplantation. *Transplantation*.

[9] Clavien PA, Harvey PRC, Strasberg SM (1992). Preservation and reperfusion injuries in liver allografts: an overview and synthesis of current studies. *Transplantation*.

[10] Noack K, Bronk SF, Kato A, Gores GJ (1993). The greater vulnerability of bile duct cells to reoxygenation injury than to anoxia: implications for the pathogenesis of biliary strictures after liver transplantation. *Transplantation*.

[11] de Oliveira ML, Jassem W, Valente R (2011). Biliary complications after liver transplantation using grafts from donors after cardiac death. *Annals of Surgery*.

[12] Heidenhain C, Pratschke J, Puhl G (2010). Incidence of and risk factors for ischemic-type biliary lesions following orthotopic liver transplantation. *Transplant International*.

[13] Op den Dries S, Sutton ME, Lisman T, Porte RJ (2011). Protection of bile ducts in liver transplantation: looking beyond ischemia. *Transplantation*.

[14] Zhai Y, Busuttil RW, Kupiec-Weglinski JW (2011). Liver ischemia and reperfusion injury: new insights into mechanisms of innate-adaptive immune-mediated tissue inflammation. *American Journal of Transplantation*.

[15] Cursio R, Gugenheim J, Ricci JE (1999). A caspase inhibitor fully protects rats against lethal normothermic liver ischemia by inhibition of liver apoptosis. *The FASEB Journal*.

[16] Cursio R (2002). *Liver ischemia-reperfusion injury in the rat: role of nonparenchymal liver cells*.

[17] Jaeschke H, Farhood A (2002). Kupffer cell activation after no-flow ischemia versus hemorrhagic shock. *Free Radical Biology and Medicine*.

[18] McKeown CMB, Edwards V, Phillips MJ, Harvey PRC, Petrunka CN, Strasberg SM (1988). Sinusoidal lining cell damage: the critical injury in cold preservation of liver allografts in the rat. *Transplantation*.

[19] Cursio R, Gugenheim J, Panaia-Ferrari P (1998). Improvement of normothermic rat liver ischemia/reperfusion by muramyl dipeptide. *Journal of Surgical Research*.

[20] Feng L, Pang L, Guo Y (2009). Hypoxia/reoxygenation up-regulates death receptor expression and enhances apoptosis in human biliary epithelial cells. *Life Sciences*.

[21] Stapleton GN, Hickman R, Terblanche J (1998). Blood supply of the right and left hepatic ducts. *The British Journal of Surgery*.

[22] Vellar ID (2001). Preliminary study of the anatomy of the venous drainage of the intrahepatic and extrahepatic bile ducts and its relevance to the practice of hepatobiliary surgery. *The Australian and New Zealand Journal of Surgery*.

[23] Vellar ID (1999). The blood supply of the biliary ductal system and its relevance to vasculobiliary injuries following cholecystectomy. *The Australian and New Zealand Journal of Surgery*.

[24] Kanno N, LeSage G, Glaser S, Alvaro D, Alpini G (2000). Functional heterogeneity of the intrahepatic biliary epithelium. *Hepatology*.

[25] Gaudio E, Onori P, Pannarale L, Alvaro D (1996). Hepatic microcirculation and peribiliary plexus in experimental biliary cirrhosis: a morphological study. *Gastroenterology*.

[26] Ludwig J (1987). New concepts in biliary cirrhosis. *Seminars in Liver Disease*.

[27] Ludwig J, Ritman EL, LaRusso NF, Sheedy PF, Zumpe G (1998). Anatomy of the human biliary system studied by quantitative computer- aided three-dimensional imaging techniques. *Hepatology*.

[28] Schaffner F, Popper H (1961). Electron microscopic studies of normal and proliferated bile ductules. *The American Journal of Pathology*.

[29] Carruthers JS, Steiner JW (1961). Studies on the fine structure of proliferated bile ductules—II. Changes of the ductule-connective tissue envelope relationship. *Canadian Medical Association Journal*.

[30] Yamamoto K, Phillips MJ (1984). A hitherto unrecognized bile ductular plexus in normal rat liver. *Hepatology*.

[31] Northover JMA, Terblanche J (1979). A new look at the arterial supply of the bile duct in man and its surgical implications. *The British Journal of Surgery*.

[32] Maguire D, Rela M, Heaton ND (2002). Biliary complications after orthotopic liver transplantation. *Transplantation Reviews*.

[33] Buis CI, Hoekstra H, Verdonk RC, Porte RJ (2006). Causes and consequences of ischemic-type biliary lesions after liver transplantation. *Journal of Hepato-Biliary-Pancreatic Surgery*.

[34] Ludwig J, Batts KP, MacCarty RL (1992). Ischemic cholangitis in hepatic allografts. *Mayo Clinic Proceedings*.

[35] Cameron AM, Busuttil RW (2005). Ischemic cholangiopathy after liver transplantation. *Hepatobiliary and Pancreatic Diseases International*.

[36] Moench C, Moench K, Lohse AW, Thies J, Otto G (2003). Prevention of ischemic-type biliary lesions by arterial back-table pressure perfusion. *Liver Transplantation*.

[37] Torras J, Lladó L, Figueras J (1999). Biliary tract complications after liver transplantation: type, management, and outcome. *Transplantation Proceedings*.

[38] Lee HW, Suh KS, Shin WY (2007). Classification and prognosis of intrahepatic biliary stricture after liver transplantation. *Liver Transplantation*.

[39] Abt P, Crawford M, Desai N, Markmann J, Olthoff K, Shaked A (2003). Liver transplantation from controlled non-heartbeating donors: an increased incidence of biliary complications. *Transplantation*.

[40] Baccarani U, Isola M, Adani GL (2010). Steatosis of the hepatic graft as a risk factor for post-transplant biliary complications. *Clinical Transplantation*.

[41] Baccarani U, Adani GL, Lorenzin D, Donini A, Risaliti A (2010). The role of steatosis of the liver graft in the development of post-transplant biliary complications. *Transplant International*.

[42] Polack WG, Porte RJ (2006). The sequence of revascularization in liver transplantation: it does make a difference. *Liver Transplantation*.

[43] Canelo R, Hakim NS, Ringe B (2003). Experience with hystidine tryptophan ketoglutarate versus University Wisconsin preservation solutions in transplantation. *International Surgery*.

[44] Pirenne J, van Gelder F, Coosemans W (2001). Type of donor aortic preservation solution and not cold ischemia time is a major determinant of biliary strictures after liver transplantation. *Liver Transplantation*.

[45] Sankary HN, McChesney L, Frye E, Cohn S, Foster P, Williams J (1995). A simple modification in operative technique can reduce the incidence of nonanastomotic biliary strictures after orthotopic liver transplantation. *Hepatology*.

[46] Li S, Stratta RJ, Langnas AN (1992). Diffuse biliary tract injury after orthotopic liver transplantation. *American Journal of Surgery*.

[47] Pirenne J, Monbaliu D, Aerts R (2009). Biliary strictures after liver transplantation: risk factors and Prevention by donor treatment with epoprostenol. *Transplantation Proceedings*.

[48] Durand F, Renz JF, Alkofer B (2008). Report of the Paris consensus meeting on expanded criteria donors in liver transplantation. *Liver Transplantation*.

[49] Haller GW, Langrehr JM, Blumhardt G (1995). Factors relevant to the development of primary dysfunction in liver allografts. *Transplantation Proceedings*.

[50] Ploeg RJ, D’Alessandro AM, Knechtle SJ (1993). Risk factors for primary dysfunction after liver transplantation—a multivariate analysis. *Transplantation*.

[51] Yska MJ, Buis CI, Monbaliu D (2008). The role of bile salt toxicity in the pathogenesis of bile duct injury after non-heart-beating porcine liver transplantation. *Transplantation*.

[52] Foley DP, Fernandez LA, Leverson G (2005). Donation after cardiac death: the University of Wisconsin experience with liver transplantation. *Annals of Surgery*.

[53] Trauner M, Meier PJ, Boyer JL (1998). Mechanisms of disease: molecular pathogenesis of cholestasis. *The New England Journal of Medicine*.

[54] Geuken E, Visser D, Kuipers F (2004). Rapid increase of bile salt secretion is associated with bile duct injury after human liver transplantation. *Journal of Hepatology*.

[55] Hertl M, Harvey PRC, Swanson PE (1995). Evidence of preservation injury to bile ducts by bile salts in the pig and its prevention by infusions of hydrophilic bile salts. *Hepatology*.

[56] Hoekstra H, Porte RJ, Tian Y (2006). Bile salt toxicity aggravates cold ischemic injury of bile ducts after liver transplantation in *Mdr*2 + /− mice. *Hepatology*.

[57] Buis CI, Geuken E, Visser DS (2009). Altered bile composition after liver transplantation is associated with the development of nonanastomotic biliary strictures. *Journal of Hepatology*.

[58] Gugenheim J, Samuel D, Reynes M, Bismuth H (1990). Liver transplantation across ABO blood group barriers. *The Lancet*.

[59] Sebagh M, Farges O, Kalil A, Samuel D, Bismuth H, Reynes M (1995). Sclerosing cholangitis following human orthotopic liver transplantation. *The American Journal of Surgical Pathology*.

[60] Sanchez-Urdazpal L, Batts KP, Gores GJ (1993). Increased bile duct complications in liver transplantation across the ABO barrier. *Annals of Surgery*.

[61] Moench C, Uhrig A, Lohse AW, Otto G (2004). CC chemokine receptor 5Δ32 polymorphism—a risk factor for ischemic-type biliary lesions following orthotopic liver transplantation. *Liver Transplantation*.

[62] Sanchez-Urdazpal L, Sterioff S, Janes C, Schwerman L, Rosen C, Krom RAF (1991). Increased bile duct complications in ABO incompatible liver transplant recipients. *Transplantation Proceedings*.

[63] Oguma S, Belle S, Starzl TE, Demetris AJ (1989). A histometric analysis of chronically rejected human liver allografts: insights into the mechanisms of bile duct loss: direct immunologic and ischemic factors. *Hepatology*.

[64] Matsumoto Y, McCaughan GW, Painter DM, Bishop GA (1993). Evidence that portal tract microvascular destruction precedes bile duct loss in human liver allograft rejection. *Transplantation*.

[65] Wu J, Ye S, Xu X, Xie H, Zhou L, Zheng S (2011). Recipient outcomes after ABO-incompatible liver transplantation: a systematic review and meta-analysis. *PLoS ONE*.

[66] Langrehr JM, Schneller A, Neuhaus R, Vogl T, Hintze R, Neuhaus P (1998). Etiologic factors and incidence of ischemic-type biliary lesions (ITBL) after liver transplantation. *Langenbeck’s Archives of Surgery*.

[67] Rull R, Garcia Valdecasas JC, Grande L (2001). Intrahepatic biliary lesions after orthotopic liver transplantation. *Transplant International*.

[68] Scotte M, Dousset B, Calmus Y, Conti F, Houssin D, Chapuis Y (1994). The influence of cold ischemia time on biliary complications following liver transplantation. *Journal of Hepatology*.

[69] Evans PC, Coleman N, Wreghitt TG, Wight DGD, Alexander GJM (1999). Cytomegalovirus infection of bile duct epithelial cells, hepatic artery and portal venous endothelium in relation to chronic rejection of liver grafts. *Journal of Hepatology*.

[70] Lautenschlager I, Höckerstedt K, Taskinen E (2003). Histologic findings associated with CMV infection in liver transplantation. *Transplantation Proceedings*.

[71] Halme L, Höckerstedt K, Lautenschlager I (2003). Cytomegalovirus infection and development of biliary complications after liver transplantation. *Transplantation*.

[72] Hoekstra H, Buis CI, Verdonk RC (2009). Is Roux-en-Y choledochojejunostomy an independent risk factor for nonanastomotic biliary strictures after liver transplantation?. *Liver Transplantation*.

[73] op den Dries S, Buis CI, Adelmeijer J (2011). The combination of primary sclerosing cholangitis and CCR5-Δ32 in recipients is strongly associated with the development of nonanastomotic biliary strictures after liver transplantation. *Liver International*.

[74] Rustgi VK, Marino G, Halpern MT, Johnson LB, Umana WO, Tolleris C (2002). Role of gender and race mismatch and graft failure in patients undergoing liver transplantation. *Liver Transplantation*.

[75] Ten Hove WR, Korkmaz KS, op den Dries S (2011). Matrix metalloproteinase 2 genotype is associated with nonanastomotic biliary strictures after orthotopic liver transplantation. *Liver International*.

[76] Buis CI, Verdonk RC, van der Jagt EJ (2007). Nonanastomotic biliary strictures after liver transplantation, part 1: radiological features and risk factors for early vs. late presentation. *Liver Transplantation*.

[77] Busquets J, Figueras J, Serrano T (2002). Postreperfusion biopsy changes predict biliary complications after liver transplantation. *Transplantation Proceedings*.

[78] Jaeschke H (1998). Mechanisms of reperfusion injury after warm ischemia of the liver. *Journal of Hepato-Biliary-Pancreatic Surgery*.

[79] Corradini SG, Micheletta F, Natoli S (2005). High preoperative recipient plasma 7*β*-hydroxycholesterol is associated with initial poor graft function after liver transplantation. *Liver Transplantation*.

[80] Jaeschke H (1991). Reactive oxygen and ischemia/reperfusion injury of the liver. *Chemico-Biological Interactions*.

[81] Ballatori N, Truong AT (1992). Glutathione as a primary osmotic driving force in hepatic bile formation. *The American Journal of Physiology*.

[82] Koeppel TA, Trauner M, Mennone A, Arrese M, Rios-Velez L, Boyer JL (1998). Role of glutathione in hepatic bile formation during reperfusion after cold ischemia of the rat liver. *Journal of Hepatology*.

[83] Accatino L, Pizarro M, Solís N, Arrese M, Koenig CS (2003). Bile secretory function after warm hepatic ischemia-reperfusion injury in the rat. *Liver Transplantation*.

[84] Accatino L, Figueroa C, Pizarro M, Solís N (1995). Enhanced biliary excretion of canalicular membrane enzymes in estrogen-induced and obstructive cholestasis, and effects of different bile acids in the isolated perfused rat liver. *Journal of Hepatology*.

[85] Celli A, Que FG, Gores GJ, LaRusso NF (1998). Glutathione depletion is associated with decreased Bcl-2 expression and increased apoptosis in cholangiocytes. *The American Journal of Physiology*.

[86] Wang Z, Zhou J, Lin J, Wang Y, Lin Y, Li X (2011). RhGH attenuates ischemia injury of intrahepatic bile ducts relating to liver transplantation. *Journal of Surgical Research*.

[87] Martinez-Mier G, Toledo-Pereyra LH, Ward PA (2000). Adhesion molecules in liver ischemia and reperfusion. *Journal of Surgical Research*.

[88] Fondevila C, Busuttil RW, Kupiec-Weglinski JW (2003). Hepatic ischemia/reperfusion injury—a fresh look. *Experimental and Molecular Pathology*.

[89] Popper H (1990). The relation of mesenchymal cell products to hepatic epithelial systems. *Progress in Liver Diseases*.

[90] Simpson KJ, Lukacs NW, Colletti L, Strieter RM, Kunkel SL (1997). Cytokines and the liver. *Journal of Hepatology*.

[91] Carrasco L, Sanchez-Bueno F, Sola J (1996). Effects of cold ischemia time on the graft after orthotopic liver transplantation: a bile cytological study. *Transplantation*.

[92] Kubota K, Ericzon BG, Barkholt L, Reinholt FP (1989). Bile cytology in orthotopic liver transplantation. *Transplantation*.

[93] Oldhafer KJ, Gubernatis G, Ringe B, Pichlmayr R (1990). Experience with bile cytology after liver transplantation. *Transplantation Proceedings*.

[94] Kubota K, Ericzon BG, Reinholt FP (1992). The correlation between cytological patterns in bile and histological findings in liver transplantation. *Transplantation*.

[95] Sheridan JP, Marsters SA, Pitti RM (1997). Control of TRAIL-induced apoptosis by a family of signaling and decoy receptors. *Science*.

[96] Spierings DC, de Vries EG, Vellenga E (2004). Tissue distribution of the death ligand TRAIL and its receptors. *Journal of Histochemistry and Cytochemistry*.

[97] Xu W-H, Ye QF, Xia SS (2004). Apoptosis and proliferation of intrahepatic bile duct after ischemia-reperfusion injury. *Hepatobiliary and Pancreatic Diseases International*.

[98] Strazzabosco M, Spirlí C, Okolicsanyi L (2000). Pathophysiology of the intrahepatic biliary epithelium. *Journal of Gastroenterology and Hepatology*.

[99] Nathanson MH, Boyer JL (1991). Mechanisms and regulation of bile secretion. *Hepatology*.

[100] Lazaridis KN, Strazzabosco M, Larusso NF (2004). The cholangiopathies: disorders of biliary epithelia. *Gastroenterology*.

[101] Boyer JL (1996). Bile duct epithelium: frontiers in transport physiology. *American Journal of Physiology*.

[102] Masyuk AI, Marinelli RA, LaRusso NF (2002). Water transport by epithelia of the digestive tract. *Gastroenterology*.

[103] Reichen J, Simon Fr, Arias IM, Boyer JL, Fausto N (1994). Cholestasis. *The Liver: Biology and Pathobiology*.

[104] Ben-Ari Z, Pappo O, Mor E (2003). Intrahepatic cholestasis after liver transplantation. *Liver Transplantation*.

[105] Cutrin JC, Cantino D, Biasi F (1996). Reperfusion damage to the bile canaliculi in transplanted human liver. *Hepatology*.

[106] Theilmann L, Otto G, Arnold J, Gmelin K, Stiehl A (1991). Biliary secretion of bile acids, lipids, and bilirubin by the transplanted liver: a quantitative study in patients on cyclosporine. *Transplantation*.

[107] Sauer P, Stiehl A, Otto G, Theilmann L (1995). In patients with orthotopic liver transplantation, serum markers of cholestasis are unreliable indicators of biliary secretion. *Journal of Hepatology*.

[108] Corbani A, Burroughs AK (2008). Intrahepatic cholestasis after liver transplantation. *Clinics in Liver Disease*.

[109] Vajdová K, Smreková R, Kukan M, Lutterová M, Wsólová L (2000). Bile analysis as a tool for assessing integrity of biliary epithelial cells after cold ischemia-reperfusion of rat livers. *Cryobiology*.

[110] Trauner M, Meier PJ, Boyer JL (1999). Molecular regulation of hepatocellular transport systems in cholestasis. *Journal of Hepatology*.

[111] Ishii M, Washioka H, Tonosaki A, Toyota T (1991). Regional orientation of actin filaments in the pericanalicular cytoplasm of rat hepatocytes. *Gastroenterology*.

[112] Cooper JA (1987). Effects of cytochalasin and phalloidin on actin. *The Journal of Cell Biology*.

[113] Mori M (1994). Electron microscopic and new microscopic studies of hepatocyte cytoskeleton: physiological and pathological relevance. *Journal of Electron Microscopy*.

[114] Emadali A, Muscatelli-Groux B, Delom F (2006). Proteomic analysis of ischemia-reperfusion injury upon human liver transplantation reveals the protective role of IQGAP1. *Molecular and Cellular Proteomics*.

[115] Doctor RB, Dahl RH, Salter KD, Fitz JG (1999). Reorganization of cholangiocyte membrane domains represents an early event in rat liver ischemia. *Hepatology*.

[116] Lingrel JB, Kuntzweiler T (1994). Na^+^,K^+^-ATPase. *The Journal of Biological Chemistry*.

[117] Selzner M, Selzmer N, Jochum W, Graf R, Clavien PA (2007). Increased ischemic injury in old mouse liver: an ATP-dependent mechanism. *Liver Transplantation*.

[118] Simon FR, Fortune J, Iwahashi M, Gartung C, Wolkoff A, Sutherland E (1996). Ethinyl estradiol cholestasis involves alterations in expression of liver sinusoidal transporters. *American Journal of Physiology*.

[119] Angermüller S, Steinmetz I, Weber T, Czerny F, Hanisch E, Kusterer K (1997). Significant increase of Kupffer cells associated with loss of Na^+^,K^+^- ATPase activity in rat hepatic allograft rejection. *Transplantation*.

[120] Erlinger S (1982). Does Na^+^-K^+^-Atpase have any role in bile secretion?. *American Journal of Physiology*.

[121] Zheng S, Feng X, Qing D, Chen M, Dong J (2008). The tolerance time limits of biliary tracts of liver grafts subjected to warm ischemia and cold preservation: an experimental study in swine. *Transplantation Proceedings*.

[122] Agermuller S, Schunk M, Kunsterer K, Konrad T, Usadel KH (1995). Alterations of Na^+^,K^+^-ATPase activity after hypoxia and reoxygenation in the perfused rat liver: an electron microscopic cytochemical study. *Journal of Hepatology*.

[123] Benkoel L, Dodero F, Hardwigsen J (2004). Effect of ischemia-reperfusion on Na^+^,K^+^-ATPase expression in human liver tissue allograft: image analysis by confocal laser scanning microscopy. *Digestive Diseases and Sciences*.

[124] Doctor RB, Dahl RH, Salter KD, Fouassier L, Chen J, Fitz JG (2000). ATP depletion in rat cholangiocytes leads to marked internalization of membrane proteins. *Hepatology*.

[125] Jaeschke H, Gores GJ, Cederbaum AI, Hinson JA, Pessayre D, Lemasters JJ (2002). Mechanisms of hepatotoxicity. *Toxicological Sciences*.

[126] Alpini G, McGill JM, LaRusso NF (2002). The pathobiology of biliary epithelia. *Hepatology*.

[127] Falasca L, Tisone G, Palmieri G (2001). Protective role of tauroursodeoxycholate during harvesting and cold storage of human liver: a pilot study in transplant recipients. *Transplantation*.

[128] Faubion WA, Guicciardi ME, Miyoshi H (1999). Toxic bile salts induce rodent hepatocyte apoptosis via direct activation of Fas. *The Journal of Clinical Investigation*.

[129] Adams DH, Afford SC (2002). The role of cholangiocytes in the development of chronic inflammatory liver disease. *Frontiers in Bioscience*.

[130] Fischer R, Cariers A, Reinehr R, Häussinger D (2002). Caspase 9-dependent killing of hepatic stellate cells by activated Kupffer cells. *Gastroenterology*.

[131] Greim H, Trülzsch D, Roboz J (1972). Mechanism of cholestasis—5. Bile acids in normal rat livers and in those after bile duct ligation. *Gastroenterology*.

[132] Oumi M, Yamamoto T (2000). A scanning electron microscope study on the effects of different bile salts on the epithelial lining of jejunal mucosa. *Medical Electron Microscopy*.

[133] Benedetti A, Alvaro D, Bassotti C (1997). Cytotoxicity of bile salts against biliary epithelium: a study in isolated bile ductule fragments and isolated perfused rat liver. *Hepatology*.

[134] Sokol RJ, Winklhofer-Roob BM, Devereaux MW, McKim JM (1995). Generation of hydroperoxides in isolated rat hepatocytes and hepatic mitochondria exposed to hydrophobic bile acids. *Gastroenterology*.

[135] Rodrigues CMP, Fan G, Wong PY, Kren BT, Steer CJ (1998). Ursodeoxycholic acid may inhibit deoxycholic acid-induced apoptosis by modulating mitochondrial transmembrane potential and reactive oxygen species production. *Molecular Medicine*.

[136] Beuers U, Hohenester S, de Buy Wenniger LJ, Kremer AE, Jansen PL, Elferink RP (2010). The biliary HCO_3_ 
^−^ umbrella: a unifying hypothesis on pathogenetic and therapeutic aspects of fibrosing cholangiopathies. *Hepatology*.

[137] Guimbellot JS, Fortenberry JA, Siegal GP (2008). Role of oxygen availability in CTFR expression and function. *American Journal of the Respiratory Cell and Mololecular Biololgy*.

[138] Tian F, Cheng L, Li D (2011). Downregulation of mucins in graft bile ducts after liver transplantation in rats. *Transplantation*.

[139] Campion JP, Porchet N, Aubert JP, L’Helgoualc’h A, Clement B (1995). UW-preservation of cultured human gallbladder epithelial cells: phenotypic alterations and differential mucin gene expression in the presence of bile. *Hepatology*.

[140] Sasaki M, Ikeda H, Nakanuma Y (2007). Expression profiles of MUC mucins and trefoil factor family (TFF) peptides in the intrahepatic biliary system: physiological distribution and pathological significance. *Progress in Histochemistry and Cytochemistry*.

[141] Urbani L, Mazzoni A, Bianco I (2008). The role of immunomodulation in ABO-incompatible adult liver transplant recipients. *Journal of Clinical Apheresis*.

[142] Hanto DW, Fecteau AH, Alonso MH, Valente JF, Whiting JF (2003). ABO-incompatible liver transplantation with no immunological graft losses using total plasma exchange, splenectomy, and quadruple immunosuppression: evidence for accomodation. *Liver Transplantation*.

[143] Okada Y, Jinno K, Moriwaki S (1988). Blood group antigens in the intrahepatic biliary tree—I. Distribution in the normal liver. *Journal of Hepatology*.

[144] Nakanuma Y, Sasaki M (1989). Expression of blood group-related antigens in the intrahepatic biliary tree and hepatocytes in normal livers and various hepatobiliary diseases. *Hepatology*.

[145] Rapaport FT, Dausset J (1987). Activity of the ABO antigen system as a determinant of histocompatibility in human transplantation. *Transplantation Proceedings*.

[146] Batts KP, Wang X (1998). Recurrence of primary biliary cirrhosis, autoimmune cholangitis and primary sclerosing cholangitis after liver transplantation. *Clinics in Liver Disease*.

[147] Vierling JM, Fennell RH (1985). Histopathology of early and late human hepatic allograft rejection: evidence of progressive destruction of interlobular bile ducts. *Hepatology*.

[148] Wiesner RH, Ludwig J, Krom RAF, Hay JE, van Hoek B (1993). Hepatic allograft rejection: new developments in terminology, diagnosis, prevention, and treatment. *Mayo Clinic Proceedings*.

[149] Perkins JD, Rakela J, Sterioff S, Banks PM, Viesner RH, Krom RA (1989). Immunoistologic pattern of the portal T-lymphocyte infiltration in hepatic allograft rejection. *Mayo Clinic Proceedings*.

[150] Beuers U (1997). Effects of bile acids on hepatocellular signaling and secretion. *Yale Journal of Biology and Medicine*.

[151] Miyake H, Tazuma S, Miura H, Yamashita G, Kajiyama G (1999). Partial characterization of mechanisms of cytoprotective action of hydrophilic bile salts against hydrophobic bile salts in rats: relation to canalicular membrane fluidity and packing density. *Digestive Diseases and Sciences*.

[152] Baiocchi L, Tisone G, Russo MA (2008). TUDCA prevents cholestasis and canalicular damage induced by ischemia-reperfusion injury in the rat, modulating PKC*α*-ezrin pathway. *Transplant International*.

[153] Golling M, Zipperle S, Weimer R (1998). Chronic liver immunologic factors in ischemic type biliary lesions (ITBL) → reduced Th1 and increased Th2 response. *Langenbecks Archiv fur Chirurgie*.

[154] Campbell WL, Sheng R, Zajko AB, Abu-Elmagd K, Demetris AJ (1994). Intrahepatic biliary strictures after liver transplantation. *Radiology*.

[155] Colonna JO, Shaked A, Gomes AS (1992). Biliary strictures complicating liver transplantation: incidence, pathogenesis, management, and outcome. *Annals of Surgery*.

[156] Ludwig J, Hashimoto E, Porayko MK, Therneau TM (1996). Failed allografts and causes of death after orthotopic liver transplantation from 1985 to 1995: decreasing prevalence of irreversible hepatic allograft rejection. *Liver Transplantation and Surgery*.

[157] Demetris AJ, Murase N, Galvao FHF (1997). Analysis of chronic rejection and obliterative arteriopathy: possible contributions of donor antigen-presenting cells and lymphatic disruption. *American Journal of Pathology*.

[158] Gouw ASH, van den Heuvel MC, van den Berg AP, Slooff MJH, de Jong KP, Poppema S (2002). The significance of parenchymal changes of acute cellular rejection in predicting chronic liver graft rejection. *Transplantation*.

[159] Singh N, Dummer JS, Kusne S (1988). Infections with cytomegalovirus and other herpesviruses in 121 liver transplant recipients: transmission by donated organ and the effect of OKT3 antibodies. *The Journal of Infectious Diseases*.

[160] Mutimer D (1996). CMV infection of transplant recipients. *Journal of Hepatology*.

[161] Martelius T, Krogerus L, Hockerstedt K, Bruggeman C, Lautenschlager I (1998). Cytomegalovirus infection is associated with increased inflammation and severe bile duct damage in rat liver allografts. *Hepatology*.

[162] Dobaczewski M, Xia Y, Bujak M, Gonzales-Quesada C, Frangogiannis NG (2010). CCR5 signaling suppresses inflammation and reduces adverse remodelling of the infracted heart, mediating recruitement of regulatory T cells. *The American Journal of Pathology*.

[163] Arthur MJP, Friedman SL, Roll FJ, Bissell DM (1989). Lipocytes from normal rat liver release a neutral metalloproteinase that degrades basement membrane (type IV) collagen. *The Journal of Clinical Investigation*.

[164] Upadhya AG, Harvey RPC, Howard TK, Lowell JA, Shenoy S, Strasberg SM (1997). Evidence of a role for matrix metalloproteinases in cold preservation injury of the liver in humans and in the rat. *Hepatology*.

[165] Cursio R, Mari B, Louis K (2002). Rat liver injury after normothermic ischemia is prevented by a phosphinic matrix metalloproteinase inhibitor. *The FASEB Journal*.

[166] Defamie V, Laurens M, Patrono D (2008). Matrix metalloproteinase inhibition protects rat livers from prolonged cold ischemia-warm reperfusion injury. *Hepatology*.

[167] Corcoran ML, Hewitt RE, Kleiner DE, Stetler-Stevenson WG (1996). MMP-2: expression, activation and inhibition. *Enzyme and Protein*.

[168] Yu AE, Hewitt RE, Kleiner DE, Stetler-Stevenson WG (1996). Molecular regulation of cellular invasion-role off gelatinase A and TIMP-2. *Biochemistry and Cell Biology*.

[169] Harendza S, Lovett DH, Panzer U, Lukacs Z, Kühnl P, Stahl RAK (2003). Linked common polymorphisms in the gelatinase a promoter are associated with diminished transcriptional response to estrogen and genetic fitness. *The Journal of Biological Chemistry*.

[170] Price SJ, Greaves DR, Watkins H (2001). Identification of novel, functional genetic variants in the human matrix metalloproteinase-2 gene: role of Sp1 in allele-specific transcriptional regulation. *The Journal of Biological Chemistry*.

[171] Selzner M, Selzner N, Chen L (2009). Exaggerated up-regulation of tumor necrosis factor *α*-dependent apoptosis in the older mouse liver following reperfusion injury: targeting liver protective strategies to patient age. *Liver Transplantation*.

[172] Okaya T, Blanchard J, Schuster R (2005). Age-dependent responses to hepatic ischemia/reperfusion injury. *Shock*.

[173] Nakamura N, Nishida S, Neff GR (2005). Intrahepatic biliary strictures without hepatic artery thrombosis after liver transplantation: an analysis of 1,113 liver transplantations at a single center. *Transplantation*.

[174] Green S (1995). PPAR: a mediator of peroxisome proliferator action. *Mutation Research*.

[175] Shin T, Kuboki S, Huber N (2008). Activation of peroxisome proliferator-activated receptor-*γ* during hepatic ischemia is age-dependent. *Journal of Surgical Research*.

[176] Kuboki S, Shin T, Huber N (2008). Peroxisome proliferator-activated receptor-*γ* protects against hepatic ischemia/reperfusion injury in mice. *Hepatology*.

[177] Akahori T, Sho M, Hamada K (2007). Importance of peroxisome proliferator-activated receptor-*γ* in hepatic ischemia/reperfusion injury in mice. *Journal of Hepatology*.

[178] El-Ghoneimi A, Cursio R, Schmid-Alliana A (2007). Pentoxifylline inhibits liver expression of tumor necrosis factor alpha mRNA following normothermic ischemia-reperfusion. *HPB*.

[179] El-Ghoneimi A, Cursio R, Schmid-Alliana A (2007). Inhibition of tumor necrosis factor alpha gene transcription by pentoxifylline reduces normothermic liver ischemia-reperfusion injury in rats. *Transplantation Proceedings*.

[180] Hilmer SN, Cogger VC, Le Couteur DG (2007). Basal activity of kupffer cells increases with old age. *Journals of Gerontology A *.

[181] de la Fuente M, Hernanz A, Guayerbas N, Alvarez P, Alvarado C (2004). Changes with age in peritoneal macrophage functions. Implication of leukocytes in the oxidative stress of senescence. *Cellular and Molecular Biology*.

[182] Kroemer G, Petit P, Zamzami N, Vayssiere JL, Mignotte B (1995). The biochemistry of programmed cell death. *The FASEB Journal*.

[183] Briceño J, Marchal T, Padillo J, Solórzano G, Pera C (2002). Influence of marginal donors on liver preservation injury. *Transplantation*.

[184] Piratvisuth T, Tredger JM, Hayllar KA, Williams R (1995). Contribution of true cold and rewarming ischemia times to factors determining outcome after orthotopic liver transplantation. *Liver Transplantation and Surgery*.

[185] Hoofnagle JH, Lombardero M, Zetterman RK (1996). Donor age and outcome of liver transplantation. *Hepatology*.

[186] Feng S, Goodrich NP, Bragg-Gresham JL (2006). Characteristics associated with liver graft failure: the concept of a donor risk index. *American Journal of Transplantation*.

[187] Padbury RTA, Attard A, Mirza DF (1994). Extended preservation of the liver with UW solution—is it justifiable?. *Transplantation*.

[188] Feller RB, Waugh RC, Selby WS, Dolan PM, Sheil AGR, Mccaughan GW (1996). Biliary strictures after liver transplantation: clinical picture, correlates and outcomes. *Journal of Gastroenterology and Hepatology*.

[189] Merion RM, Pelletier SJ, Goodrich N, Englesbe MJ, Delmonico FL (2006). Donation after cardiac death as a strategy to increase deceased donor liver availability. *Annals of Surgery*.

[190] Jay CL, Lyuksemburg V, Ladner DP (2011). Ischemic cholangiopathy after controlled donation after cardiac death liver transplantation: a meta-analysis. *Annals of Surgery*.

[191] Abt PL, Desai NM, Crawford MD (2004). Survival following liver transplantation from non-heart-beating donors. *Annals of Surgery*.

[192] de Vera ME, Lopez-Solis R, Dvorchik I (2009). Liver transplantation using donation after cardiac death donors: long-term follow-up from a single center. *American Journal of Transplantation*.

[193] Chan EY, Olson LC, Kisthard JA (2008). Ischemic cholangiopathy following liver transplantation from donation after cardiac death donors. *Liver Transplantation*.

[194] Skaro AI, Jay CL, Baker TB (2009). The impact of ischemic cholangiopathy in liver transplantation using donors after cardiac death: the untold story. *Surgery*.

[195] Fondevila C, Hessheimer AJ, Ruiz A (2007). Liver transplant using donors after unexpected cardiac death: novel presentation protocol and acceptance criteria. *American Journal of Transplantation*.

[196] Sibulesky L, Nguyen JH (2011). Update on biliary strictures in liver transplants. *Transplantation Proceedings*.

[197] Sherlock S (1983). Acute fatty liver of pregnancy and the microvesicular fat diseases. *Gut*.

[198] Koneru B, Dikdan G (2002). Hepatic steatosis and liver transplantation current clinical and experimental perspectives. *Transplantation*.

[199] Spitzer AL, Lao OB, Dick AAS (2010). The biopsied donor liver: incorporating macrosteatosis into high-risk donor assessment. *Liver Transplantation*.

[200] de Graaf EL, Kench J, Dilworth P (2012). Grade of deceased donor liver macrovesicular steatosis impacts graft and recipient outcomes more than the Donor Risk Index. *Journal of Gastroenterology and Hepatology*.

[201] Marsman WA, Wiesner RH, Rodriguez L (1996). Use of fatty donor liver is associated with diminished early patient and graft survival. *Transplantation*.

[202] Sun CK, Zhang XY, Zimmermann A, Davis G, Wheatley AM (2001). Effect of ischemia-reperfusion injury on the microcirculation of the steatotic liver of the Zucker rat. *Transplantation*.

[203] Onumata O, Takahashi T, Sato K, Kakita A (2000). Effects of ulinastatin on hypotension and hepatic circulation in brain dead rabbits. *Transplantation Proceedings*.

[204] Yamagami K, Hutter J, Yamamoto Y (2005). Synergistic effects of brain death and liver steatosis on the hepatic microcirculation. *Transplantation*.

[205] Campra JL, Reynolds TB, Arias IM, Jakoby WB, Popper H, Schachter D, Shafritz DA (1988). The hepatic circulation. *The Liver: Biology and Pathophysiology*.

[206] Post S, Palma P, Gonzalez AP, Rentsch M, Menger MD (1994). Timing of arterialization in liver transplantation. *Annals of Surgery*.

[207] Puhl G, Schaser KD, Pust D (2004). The delay of rearterialization after initial portal reperfusion in living donor liver transplantation significantly determines the development of microvascular graft dysfunction. *Journal of Hepatology*.

[208] Reck T, Steinbauer F, Steinbauer M (1996). Impact of arterialization on hepatic oxygen supply, tissue energy phosphates, and outcome after liver transplantation in the rat. *Transplantation*.

[209] Platz KP, Mueller AR, Schäfer C, Jahns S, Guckelberger O, Neuhaus P (1997). Influence of warm ischemia time on initial graft function in human liver transplantation. *Transplantation Proceedings*.

[210] Heidenhain C, Heise M, Jonas S (2006). Retrograde reperfusion via vena cava lowers the risk of initial nonfunction but increases the risk of ischemic-type biliary lesions in liver transplantation—a randomized clinical trial. *Transplant International*.

[211] Polak WG, Miyamoto S, Nemes BA (2005). Sequential and simultaneous revascularization in adult orthotopic piggyback liver transplantation. *Liver Transplantation*.

[212] Massarollo PCB, Mies S, Raia S (1998). Simultaneous arterial and portal revascularization in liver transplantation. *Transplantation Proceedings*.

[213] Adani GL, Rossetto A, Bresadola V, Lorenzin D, Baccarani U, de Anna D (2011). Contemporaneous portal-arterial reperfusion during liver transplantation: preliminary results. *Journal of Transplantation*.

[214] Terblanche J, Worthley CS, Spence RAJ, Krige JEJ (1990). High or low hepaticojejunostomy for bile duct strictures?. *Surgery*.

[215] Tzakis AG, Gordon RD, Shaw BW, Iwatsuki S, Starzl TE (1985). Clinical presentation of hepatic artery thrombosis after liver transplantation in the cyclosporine era. *Transplantation*.

[216] Farid WRR, de Jonge J, Slieker JC (2011). The importance of portal venous blood flow in ischemic-type biliary lesions after liver transplantation. *American Journal of Transplantation*.

[217] Lee ChY, Mangino MJ (2009). Preservation methods for kidney and liver. *Organogenesis*.

[218] Cui DX, Yin JQ, Xu WX, Chai F, Liu BL, Zhang XB (2010). Effect of different bile duct flush solutions on biliary tract preservation injury of donated livers for transplantation. *Transplantation Proceedings*.

[219] Puhl G, Olschewski P, Schöning W (2006). Low viscosity histidine-tryptophan-ketoglutarate graft flush improves subsequent extended cold storage in University of Wisconsin solution in an extracorporeal rat liver perfusion and rat liver transplantation model. *Liver Transplantation*.

[220] Welling TH, Heidt DG, Englesbe MJ (2008). Biliary complications following liver transplantation in the model for end-stage liver disease era: effect of donor, recipient, and technical factors. *Liver Transplantation*.

[221] Feng L, Zhao N, Yao X (2007). Histidine-tryptophan-ketoglutarate solution vs. University of Wisconsin solution for liver transplantation: a systematic review. *Liver Transplantation*.

[222] Demetris AJ, Fontes P, Lunz JG, Specht S, Murase N, Marcos A (2006). Wound healing in the biliary tree of liver allografts. *Cell Transplantation*.

[223] Eghtesad B, Aucejo F, Fung JJ (2006). Preservation solutions in liver transplantation: what are the options?. *Liver Transplantation*.

[224] Fridell JA, Mangus RS, Tector AJ (2009). Clinical experience with histidine-tryptophan-ketoglutarate solution in abdominal organ preservation: a review of recent literature. *Clinical Transplantation*.

[225] Erhard J, Lange R, Scherer R (1994). Comparison of histidine-tryptophan-ketoglutarate (HTK) solution versus University of Wisconsin (UW) solution for organ preservation in human liver transplantation. A prospective, randomized study. *Transplant International*.

[226] Lange R, Erhard J, Rauen U, de Groot H, Eigler FW (1997). Hepatocellular injury during preservation of human livers with UW and HTK solution. *Transplantation Proceedings*.

[227] Moench C, Otto G (2006). Ischemic type biliary lesions in histidine-tryptophan-ketoglutarate (HTK) preserved liver grafts. *The International Journal of Artificial Organs*.

[228] Mangus RS, Tector AJ, Agarwal A, Vianna R, Murdock P, Fridell JA (2006). Comparison of histidine-tryptophan-ketoglutarate solution (HTK) and University of Wisconsin solution (UW) in adult liver transplantation. *Liver Transplantation*.

[229] Avolio AW, Agnes S, Nure E (2006). Comparative evaluation of two perfusion solutions for liver preservation and transplantation. *Transplantation Proceedings*.

[230] Hatano E, Kiuchi T, Tanaka A (1997). Hepatic preservation with histidine-tryptophan-ketoglutarate solution in living-related and cadaveric liver transplantation. *Clinical Science*.

[231] Kinoshita K, Ikai I, Gomi T (1998). Exposure of hepatic simusoidal mononuclear cells to UW solution *in situ* but not *ex vivo* induces apoptosis. *Journal of Hepatology*.

[232] Testa G, Malagó M, Nadalin S (2003). Histidine-tryptophan-ketoglutarate versus University of Wisconsin solution in living donor liver transplantation: results of a prospective study. *Liver Transplantation*.

[233] Chan SC, Liu CL, Lo CM, Fan ST (2004). Applicability of histidine-tryptophan-ketoglutarate solution in right lobe adult-to-adult live donor liver transplantation. *Liver Transplantation*.

[234] Janßen H, Janßen PHE, Broelsch CE (2004). UW is superior to Celsior and HTK in the protection of human liver endothelial cells against preservation injury. *Liver Transplantation*.

[235] Abrahamse SL, van Runnard Heimel P, Hartman RJ, Chamuleau RAFM, van Gulik TM (2003). Induction of necrosis and DNA fragmentation during hypothermic preservation of hepatocytes in UW, HTK, and celsior solutions. *Cell Transplantation*.

[236] Straatsburg IH, Abrahamse SL, Song SW, Hartman RJ, van Gulik TM (2002). Evaluation of rat liver apoptotic and necrotic cell death after cold storage using UW, HTK, and Celsior. *Transplantation*.

[237] Walcher F, Marzi I, Schafer W, Flecks U, Larsen R (1995). Undissolved particles in UW solution cause microcirculatory disturbances after liver transplantation in the rat. *Transplant International*.

[238] Tullius SG, Filatenkow A, Horch D (2002). Accumulation of crystal deposits in abdominal organs following perfusion with defrosted University of Wisconsin solutions. *American Journal of Transplantation*.

[239] Feng XN, Xu X, Zheng SS (2006). Current status and perspective of liver preservation solutions. *Hepatobiliary and Pancreatic Diseases International*.

[240] Fung JJ, Eghtesad B, Patel-Tom K (2007). Using livers from donation after cardiac death donors—a proposal to protect the true Achilles heel. *Liver Transplantation*.

[241] Mangus RS, Fridell JA, Vianna RM (2008). Comparison of histidine-tryptophan-ketoglutarate solution and University of Wisconsin solution in extended criteria liver donors. *Liver Transplantation*.

[242] Gong J, Lao XJ, Wang XM, Long G, Jiang T, Chen S (2008). Preservation of non-heart-beating donor livers in extracorporeal liver perfusion and histidine-trytophan-ketoglutarate solution. *World Journal of Gastroenterology*.

[243] Lang R, He Q, Jin ZK, Han DD, Chen DZ (2009). Urokinase perfusion prevents intrahepatic ischemic-type biliary lesion in donor livers. *World Journal of Gastroenterology*.

[244] Gok MA, Shenton BK, Buckley PE (2003). How to improve the quality of kidneys from non-heart-beating donors: a randomised controlled trial of thrombolysis in non-heart-beating donors. *Transplantation*.

